# Barotraumatisme du sinus frontal à propos d’un cas et revue de la littérature

**DOI:** 10.11604/pamj.2016.24.272.5742

**Published:** 2016-07-27

**Authors:** Azeddine Lachkar, Ahmed Aabach, Fahd Elayoubi, Rachid Ghailan

**Affiliations:** 1 Service d’Oto-Rhino-Laryngologie et Chirurgie Cervico-Faciale, CHU Mohammed VI, Oujda, Maroc

**Keywords:** Barotraumatisme, sinus frontal, plongée, Barotrauma, frontal sinus, diving

## Abstract

Le barotraumatisme sinusien est le deuxième en fréquence après celui de l'oreille moyenne. Le sinus frontal est le plus touché. C'est une pathologie rare et spécifique. Nous rapportons le cas d'un patient de 26 ans présentant suite à une plongée des douleurs frontales gauche intense avec épistaxis homolatérale. Le scanner a montré un hémosinus frontal gauche. L'évolution était bonne après traitement. Les barotraumatismes du sinus frontal sont des accidents liés aux variations de la pression ambiante. L'épistaxis est un signe de gravité. Le scanner a un intérêt dans la recherche des facteurs favorisants et la surveillance. Le traitement vise à supprimer la symptomatologie et la cause favorisante. Les barotraumatismes sinusiens constituent un accident rare, les complications orbitaires et encéphaliques sont exceptionnelles. Leur traitement curatif se confond avec celui de la pathologie causale.

## Abstract (en)

Sinus barotrauma is the second most frequently reported injury after middle ear barotrauma. The front sinus is the most common site affected. This is a rare and specific pathology. We here report the case of a 26-year-old patient having severe left frontal pain with ipsilateral epistaxis after a dive. CT scan showed left frontal hemosinus. Patient evolution was good after therapy. Frontal sinus barotraumas are accidents related to the variations in environmental pressure. Epistaxis is a sign of serious health condition, CT scanner plays a role in evaluating potential predisposing factors and establishing monitoring procedures. Patient treatment aims to relieve symptoms and to remove predisposing factors. Frontal sinus barotraumas are a rare injury ; orbital and encephalic complications are exceptional. Their treatment coincides with that of their causal pathology.

## Introduction

Le barotraumatisme est la forme la plus fréquente de lésions liées à la plongée sous marine. Ces accidents peuvent avoir des conséquences graves nécessitant une prise en charge spécialisée. Le barotraumatisme sinusien est le deuxième en fréquence après celui de l'oreille moyenne. Le sinus frontal est le plus touché [1]. Nous rapportons le cas d'un barotraumatisme du sinus frontal chez un amateur de plongée.

## Patient et observation

**Figure 1:**
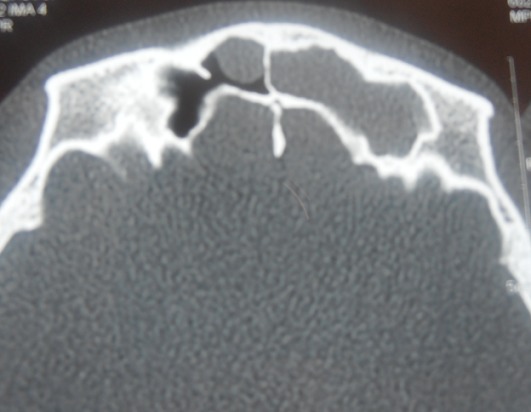
Scanner en coupe axiale montrant un comblement total du sinus frontal gauche

**Figure 2:**
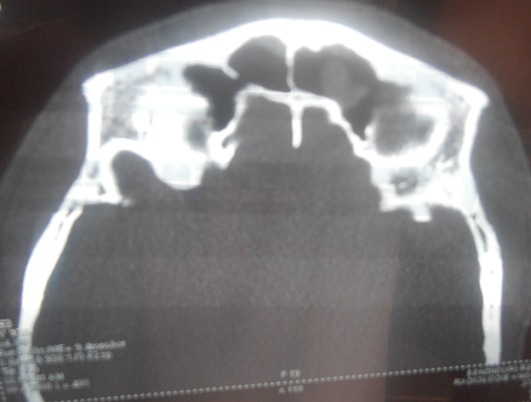
Scanner en coupe axiale montrant la régression de l’opacité sinusienne

Il s'agit d'un patient âgé de 26 ans sans antécédents pathologiques, qui a présenté suite à une plongée au moment de monté des douleurs frontales localisées à gauche en coup de poignard avec épistaxis homolatérale de faible abondance et sensation de plénitude d'oreille gauche. L'absence d'amélioration malgré un traitement symptomatique de cinq jours a motivé le patient de nous consulter. L'endoscopie nasale a objectivé une muqueuse nasale oedématiée légèrement inflammatoire avec des secrétions sanguinolentes. Une TDM nasosinusienne a été demandée et a mis en évidence une opacité diffuse comblant le sinus frontal gauche (Figure 1). L'audiométrie et l'impedancemétrie étaient normaux. Un traitement a été prescrit comportant une fluoroquinolone, un corticoïde, un antalgique palier II, et un lavage des fosses nasales. L'évolution était favorable en dix jours. Un scanner de contrôle a été demandé trois semaines après et a objectivé une régression de l'opacité sinusienne (Figure 2).

## Discussion

Les barotraumatismes du sinus frontal sont des accidents rares liés aux variations de la pression ambiante, qui peuvent survenir au cours de la pratique de la plongée subaquatique. Ces phénomènes pathologiques peuvent aussi bien se produire à la descente qu'à la remontée. Le sinus frontal est une cavité aréique creusée dans l'épaisseur de l'os frontal, tapissée par une muqueuse respiratoire. Il communique avec la fosse nasale par le conduit nasofrontal. Ce dernier est long et étroit, souvent sinueux et de morphologie variable en raison de son trajet intraethmoïdal et des nombreuses variations anatomiques de cette région. Toute altération de leur fonctionnement, associée à une variation importante de la pression ambiante, retentit immédiatement sur les échanges gazeux nasosinusiens et favorise la survenue du BTS [1, 2]. Quand la pression ambiante augmente, la dépression endosinusienne relative accentue l´obstruction ostiale en renforçant la coaptation des lèvres de la muqueuse méatale oedémateuse, qui joue le rôle d´une valve unidirectionnelle et transforme le sinus en une cavité close en dépression relative: le barotraumatisme se constitue. Trois stades anatomocliniques de gravité sont décrits: 1^er^ degrè: un oedème et une hyperhémie exsudative de la muqueuse apparaissent; 2^ème^ degrè: décollement intramuqueux et épanchement séromuqueux ou sérohématique; 3^ème^ degrè: hématome sous-muqueux, puis rupture muqueuse et hémorragie intrasinusienne Quand la pression ambiante diminue, la surpression endosinusienne qui en résulte est bien tolérée, car l´ostium se laisse habituellement forcer dans ce sens. En revanche, lorsque la valve est obstructive ou s´il s´agit d´un polype endosinusien venant jouer le rôle d´une soupape interne, l´équilibre des pressions ne peut s´établir: l´air peut entrer dans le sinus mais ne peut en sortir et il se constitue un barotraumatisme «à pression inversée». La muqueuse est plaquée contre les parois par la surpression endosinusienne: il en résulte une douleur et, parfois, la rupture de la paroi osseuse dans une zone fragile, avec constitution d´un emphysème sous-cutané [1, 3]. La symptomatologie est faite d'une douleur au niveau de l'angle supéro-interne de l'orbite avec irradiation sus orbitaire, intense parfois syncopale. Une épistaxis homolatérale modérée est un signe de gravité. L'examen clinique est pauvre.

L'endoscopie nasale précise l'origine de l'épistaxis, l'aspect de la muqueuse et des secrétions nasales, ainsi que la recherche des facteurs favorisants [4]. Le scanner a un intérêt limité dans le diagnostic, car il n'existe pas de parallélisme entre la symptomatologie et les données scannographiques. Cependant il est utile dans la mise en évidence des facteurs favorisants et la surveillance de l'évolution [5]. Le traitement vise à supprimer la symptomatologie aigue et la cause éventuelle. Il s'agit de calmer la douleur par des antalgiques, rétracter la muqueuse par des vasoconstricteurs, lutter contre l'œdème par une corticothérapie orale en cure courte, prévenir l'infection par une antibiothérapie à large spectre, et faciliter le drainage naso-sinusien par des lavage au sérum physiologique. Le traitement étiologique s´appuie sur un bilan rhinosinusien complet, endoscopique, radiologique et fonctionnel. Il fait appel à des moyens instrumentaux ou chirurgicaux pour restaurer le fonctionnement sinusien en éliminant une lésion irréversible ou inaccessible à un traitement médical. La microchirurgie endonasale trouve ici un terrain de prédilection par son caractère atraumatique, sa vocation essentiellement fonctionnelle et la qualité de ses résultats. Selon les cas, elle peut se limiter à une turbinoplastie inférieure, un nettoyage du méat moyen, une méatotomie, une cure de concha bullosa obstructive, ou aller jusqu´à une infundibulotomie ou un évidement ethmoïdal pour les formes compliquées. Une septoplastie classique peut être proposée isolément ou en association avec l´un de ces gestes [5].

## Conclusion

Les barotraumatismes sinusiens constituent un accident rare, les complications orbitaires et encéphaliques sont exceptionnelles. Leur traitement curatif se confond avec celui de la pathologie causale. La prévention des barotraumatismes sinusiens repose sur un examen d'aptitude spécialisé minutieux. L'essor de la chirurgie ethmoïdale impose au rhinologiste une bonne connaissance de cette pathologie et de ses risques.
